# Revisiting the Most Stable Structures of the Benzene Dimer

**DOI:** 10.3390/ijms25158272

**Published:** 2024-07-29

**Authors:** Jiří Czernek, Jiří Brus

**Affiliations:** Institute of Macromolecular Chemistry, Czech Academy of Sciences, Heyrovsky Square 2, 162 00 Prague, Czech Republic; brus@imc.cas.cz

**Keywords:** noncovalent interactions, intermolecular stacking, interaction energy, CCSD(T), SAPT

## Abstract

The benzene dimer (BD) is an archetypal model of π∙∙∙π and C–H∙∙∙π noncovalent interactions as they occur in its cofacial and perpendicular arrangements, respectively. The enthalpic stabilization of the related BD structures has been debated for a long time and is revisited here. The revisit is based on results of computations that apply the coupled-cluster theory with singles, doubles and perturbative triples [CCSD(T)] together with large basis sets and extrapolate results to the complete basis set (CBS) limit in order to accurately characterize the three most important stationary points of the intermolecular interaction energy (Δ*E*) surface of the BD, which correspond to the tilted T-shaped (TT), fully symmetric T-shaped (FT) and slipped-parallel (SP) structures. In the optimal geometries obtained by searching extensive sets of the CCSD(T)/CBS Δ*E* data of the TT, FT and SP arrangements, the resulting Δ*E* values were −11.84, −11.34 and −11.21 kJ/mol, respectively. The intrinsic strength of the intermolecular bonding in these configurations was evaluated by analyzing the distance dependence of the CCSD(T)/CBS Δ*E* data over wide ranges of intermonomer separations. In this way, regions of the relative distances that favor BD structures with either π∙∙∙π or C–H∙∙∙π interactions were found and discussed in a broader context.

## 1. Introduction

Interactions between aromatic rings are at the heart of some important phenomena currently studied in structural biology [1], chemical synthesis [2], catalysis [3], drug delivery [4], crystal engineering [5], molecular electronics [6], materials science [7] and other fields. The two most important noncovalent binding motifs of aromatic rings feature either π∙∙∙π stacking (see reference [8] and the works cited therein) or C–H∙∙∙π [9] “T-shaped” intermolecular interactions. Importantly, the benzene dimer (BD) in its ground electronic state in the gas phase exhibits these types of interactions in low-lying regions of the potential energy surface (PES) (see the most recent description in reference [10] and in this work) and the Gibbs free energy landscape (see the debate in [11,12,13]). The BD is thus an archetypal model for both π∙∙∙π and aromatic C–H∙∙∙π interactions and has been investigated by numerous experimental and computational techniques. In particular, a global minimum (GM) of the BD PES was sought for a long time. Notable earlier experimental studies include the mass spectroscopy investigation that found a parallel displaced structure (π∙∙∙π stacked, of *C*_2h_ symmetry) to be the GM [14] and the rotational spectroscopic measurements interpreted in terms of a symmetrical top structure (C–H∙∙∙π T-shaped with *C*_2v_ symmetry) as the GM [15], while some time later, infrared spectroscopy measurements confirmed a bent T-shaped structure (of *C*_s_ symmetry) to be the GM [16]; see also references [17,18,19,20]. It should be mentioned that the BD is a quite challenging system for experiments, as even conflicting measurements of its dissociation energy are available [21,22]. At the same time, the BD is also challenging for computations, mainly due to the importance of the higher-order electron correlation (see reference [23] for background) for a proper description of some regions of the PES, which was most recently discussed in reference [10]. In general, an application of the coupled-cluster theory with singles, doubles and perturbative triples [CCSD(T)] is required to reliably characterize the stationary points of the BD PES (see reference [10] for a list of previous CCSD(T) computations on the BD). However, also, the symmetry-adapted perturbation theory (SAPT) of intermolecular interactions [24] based on the density-functional theory (DFT) description of monomers [25] provided accurate results for important configurations of the BD (see, in particular, references [18,26]), and the quantum Monte Carlo (QMC) [27] method was applied for benchmarking purposes to the aforementioned *C*_2h_ and *C*_2v_ symmetrical structures in a recent investigation [28]. Moreover, the spin-component scaled second-order Møller–Plesset (SCS MP2) method (see review [29]) was employed in the benchmark studies of the BD [10,30]. It is thus important to consistently apply the CCSD(T) method together with large basis sets to extrapolate the underlying energies of the complete basis set (CBS) limit for a highly accurate description of the geometries and their interaction energies (denoted here as Δ*E* and given in kJ/mol) of this challenging system. These CCSD(T)/CBS geometries and their Δ*E* data are presented in Section 2 for the three most important stationary points of the BD PES, namely, the tilted T-shaped (TT), fully symmetric T-shaped (FT) and slipped-parallel (SP) structures. The level of accuracy of present CCSD(T)/CBS computations is established in Section 2.1, mainly by comparison with the most recent results [10], while Section 2.2, Section 2.3 and Section 2.4 describe the regions of the PES pertinent to the TT, FT and SP configurations. This revisit of the key stationary points of the PES aims at describing several important characteristics of the BD. In particular, the distance dependence of the CCSD(T)/CBS Δ*E* data over wide ranges of intermonomer separations is characterized in terms of the same analytical model. Section 3 deals with various aspects of the enthalpic stabilization of investigated configurations. The SAPT-DFT/CBS data for the two perpendicular-like structures (TT and FT) are presented in Section 3.1 and enable description of the physical contributions that cause the tilted structure to be the GM. Importantly, the first-order SAPT contributions, that is, sums of the electrostatic and Pauli repulsion terms (see Section 4), were found to be responsible for a higher stabilization of the TT dimers than of their FT counterparts, which was confronted with an analysis recently provided by Carter-Fenk and Herbert [31]. In Section 3.2, the intrinsic strength of intermolecular interactions [32] in cofacial and T-shaped structures was compared. Significantly, only for the intermonomer separations close to the corresponding CCSD(T)/CBS Δ*E* minimal values, the intrinsic strength of C–H∙∙∙π noncovalent bonding, as present in the TT and FT configurations, was higher than in the π∙∙∙π stacked arrangement of the SP structures. This finding is related to the highly fluxional character of the BD (already, at this point, it should be noted that differences in thermal and nuclear quantum effects between T-shaped and parallel-like clusters were most recently described in reference [33]). Furthermore, this finding is related to the prevalence of π-stacked arrangements over C–H∙∙∙π motifs in supramolecular systems (see the most recent investigations: [8,34,35,36] and the works cited therein).

## 2. Results

### 2.1. Confrontation with Previous Data

The plain and augmented polarized-valence correlation-consistent X-zeta basis sets [37,38] were utilized in the most recent study of the BD [10] and in this work, respectively, to obtain the counterpoise-corrected [39] (CP) interaction energy values and optimize the geometries. In what follows, the families of these plain and augmented basis sets are abbreviated as VXZ and aVXZ, respectively, for a given cardinal number, X (thus, for instance, “VTZ” denotes the plain triple-zeta basis set and “aV5Z” denotes the augmented quintuple-zeta basis set). The focal-point approach expressed by Equation (1) (see Section 4) was employed in order to estimate the CCSD(T)/CBS Δ*E* during searches of the PES. In short, it adopts the aV5Z basis set in the computations of the Hartree–Fock (HF) and MP2 portions of a total Δ*E*, while the aVTZ basis set is used to approximate the post-MP2 contribution to the investigated Δ*E* value (details, together with references, are given in Section 4). This approach was thoroughly tested before [40,41,42] and found to perform well for the CCSD(T)/CBS Δ*E* estimation of the aforementioned TT, FT and SP configurations also, in their geometries taken from reference [10], where VXZ basis sets (up to V5Z) were used. Namely, interaction energies of −11.80, −11.26 and −11.22 kJ/mol were obtained here by applying Equation (1) to the TT, FT and SP geometries from reference [10], respectively. These values do not differ by more than 0.10 kJ/mol from the error bars of the respective CCSD(T)/CBS Δ*E* data reported in reference [10] (see Table 1). Hence, the present focal-point approach was used in the scans of the PES, which are described in Section 2.2 and Section 2.3.

**Figure 1:**
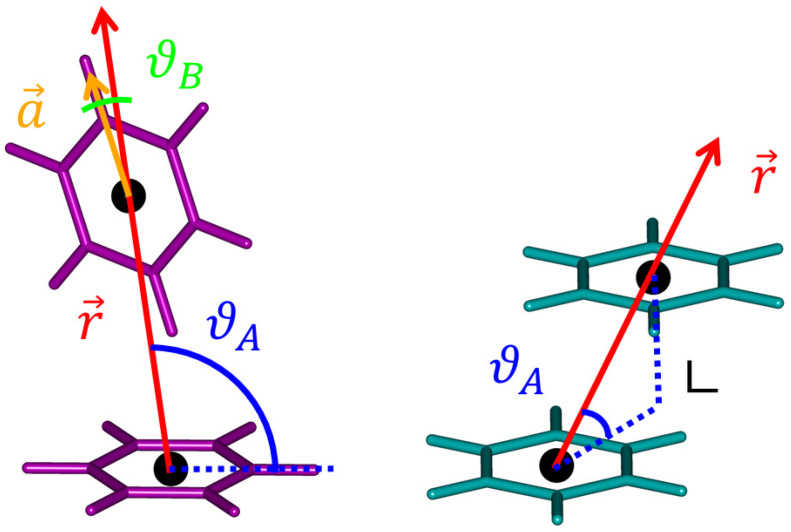
The tilted T-shaped (in magenta, to the left) and slipped-parallel (in cyan, to the right) structures of the benzene dimer, shown at the same scale. Also depicted is a schematic representation of the geometric parameters defining the respective angles $\vartheta_{A}$ and $\vartheta_{B}$.

Table 1 summarizes the main results obtained here and in references [10,18]. In the latter, highly accurate SAPT-DFT computations were performed to fit the full PES to a functional form that depended on six variables, which were defined in reference [43] and adopted in preparing coordinates in the present work also (see Section 4 for details). Results from a fit to the “pot3” potential of reference [18] are included in Table 1. The values of the distance between the centers of mass of the monomers, *R*, and of the angles defining the mutual orientation of the respective structures, $\vartheta_{A}$ and $\vartheta_{B}$ (see Figure 1), are listed together with the best estimates of the interaction energy. Specifically, the costly method expressed by Equation (2) (see Section 4) was applied in this work to the final CCSD(T)/CBS geometries to obtain their Δ*E* data. In brief, the HF component of the Δ*E* was estimated using the aV5Z basis set, and the correlation energy component was obtained from an extrapolation of the CCSD(T)/aVTZ and CCSD(T)/aVQZ data (see Section 4 for details and references). An inspection of Table 1 reveals that the present approach provided results that are almost the same as their counterparts from reference [18], with differences of about 1 pm, 1 degree and 0.1 kJ/mol for the pertinent values of the distance, angles and Δ*E*, respectively. Interestingly, the SCS-MP2/aTZ geometry optimization of the GM performed in reference [10] led to slightly smaller values of *R* and $\vartheta_{B}$ than those obtained in this work and in reference [18], but these discrepancies did not result in any significant differences in the interaction energies (see Table 1). This is likely related to a very shallow PES around the GM, which is described in the subsequent paragraph.

### 2.2. The Tilted T-Shaped Structure

An application of gradient-based optimization techniques to the BD PES is prone to issues. For example, a MP2/aVTZ gradient optimization of T-shaped structures did not lead to geometries with a correct number of imaginary frequencies (see part IIIa of the most recent study, [10], for details). In the course of this work, it was found out that a number of combinations of DFT functional and basis sets (not shown) did not correctly characterize all the aforementioned FT, TT and SP configurations at once (see also Table 1 of reference [10]). Hence, as in numerous previous investigations of the BD, the direct search approach was adopted. It established an optimal geometry from Δ*E* values computed for rigid monomers at a large number of grid points in the relevant region of the PES. It should be mentioned that the thermally averaged structure of benzene was used [44] (a small effect of the deformation energy of monomers upon the Δ*E* data is addressed in Section 3.1). It should also be mentioned that the internal coordinates as defined in reference [43] (the distance between the centers of the monomers, *R*, and the set of Euler angles) were conveniently employed in the PES searches, which are detailed below.

**Figure 2:**
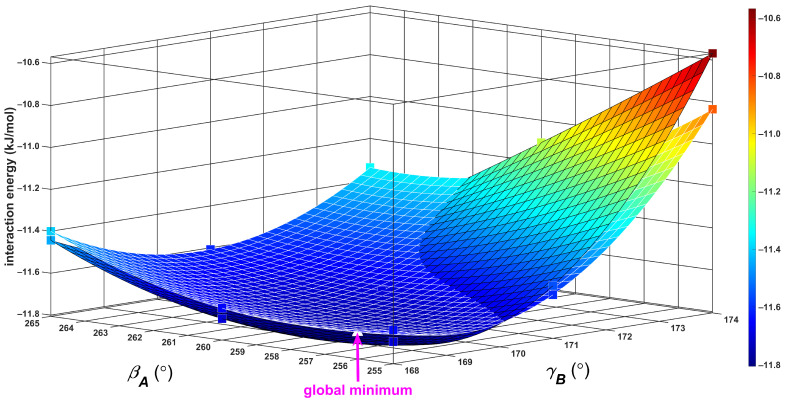
Surface plot of the intermolecular interaction energy in the region around the global minimum of the benzene dimer. The $\left(\beta_{A},\gamma_{B}\right)$ angular cuts generated by an interpolation of the $\Delta E\left(\beta_{A},\gamma_{B},R\right)$ data points for *R* values of 490.00 and 498.75 pm are shown with black and white mesh, respectively.

**Figure 3:**
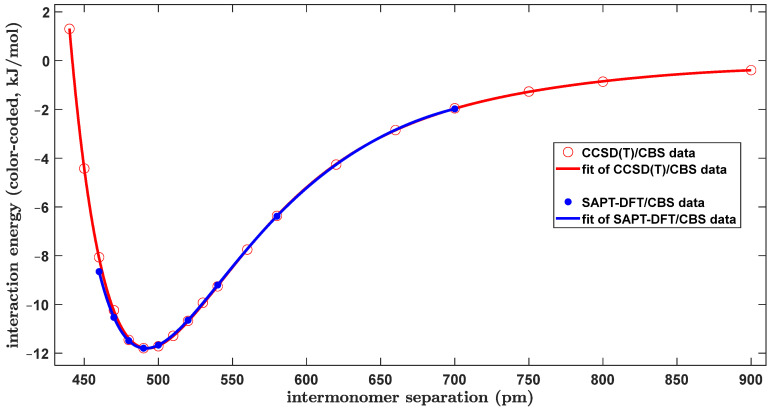
Plot of the distance dependence of the intermolecular interaction energy of the tilted T-shaped dimer of benzene.

During the initial search, a relatively small region of the PES containing the two T-shaped configurations was surveyed by the ωB97X-3c/vDZP method [45], which is a very computationally cheap yet quite reliable DFT-based approach [46]. Namely, geometries were prepared using the three-dimensional (3D) grid, which was created by independently varying the distance, *R*, and the Euler angles, $\beta_{A}$, and $\gamma_{B}$ from the aforementioned study [43]. The canonical T-shaped structure, due to its *C*_2v_ symmetry, had $\beta_{A}$ and $\gamma_{B}$ values constrained at 180° and 270°, respectively. Furthermore, a minimum of the tilted structure was expected at $\beta_{A}$ and $\gamma_{B}$ values of about 170° and 260°, respectively, as it was realized that these values would correspond to $\vartheta_{A}$ and $\vartheta_{B}$ values of 100° and 10°, respectively (see Table 1). Hence, a uniformly spaced grid was chosen to cover the $\langle 168{}^{\circ};\;180{}^{\circ}\rangle$ and $\langle 250{}^{\circ};\;270{}^{\circ}\rangle$ intervals of the $\beta_{A}$ and $\gamma_{B}$ values, respectively, together with a wide $\langle 490;\;525\rangle$ pm interval of *R* values. The ωB97X-3c/vDZP interaction energies at this 3D grid span an interval from about −12.3 to −8.7 kJ/mol. These $\Delta E\left(\beta_{A},\gamma_{B},R\right)$ data are visualized in Supporting Information Figure S1, together with a global minimum found by 3D interpolation (see Section 4 for technical details) at a point lying around $\beta_{A}=170{}^{\circ}$; $\gamma_{B}=256{}^{\circ}$; R=500 pm, with an ΔE of about −12.4 kJ/mol and thus reasonably close to the results of the highly demanding calculations shown in Table 1. Consequently, various surface plots of the ωB97X-3c/vDZP ΔE values were inspected to then properly choose even smaller and tighter grids for the CCSD(T)/CBS computations of tilted T-shaped geometries. Numerous such computations had to be performed anyway in order to be able to bracket the GM in a grid suitable for an evaluation of the resulting $\left(\beta_{A},\gamma_{B},R\right)$ values, which are presented in Table 1. The coordinates of this final grid are listed in Supporting Information Table S1, together with the associated CCSD(T)/CBS Δ*E* values, and these 27 $\Delta E\left(\beta_{A},\gamma_{B},R\right)$ data points and a position of the GM are visualized in Figure S2. A subset of this grid is used in Figure 2 to illustrate a flat and intricate character of the PES around the GM, which was, of course, expected [10]. Namely, two $\Delta E\left(\beta_{A},\gamma_{B}\right)$ surfaces, which were obtained for the *R* distance fixed at values differing by almost nine pm (see Figure 2), are shown. There is only a small variation in the ΔE on these surfaces (up to ca. 0.27 kJ/mol at the point where $\beta_{A}=174{}^{\circ}$ and $\gamma_{B}=255{}^{\circ}$). Interestingly, the two surfaces even intersected in the investigated region. An inspection of Figure 2 further reveals that the Δ*E* data in this region are also only mildly sensitive to changes in either the $\beta_{A}$ and $\gamma_{B}$ angular coordinates. Hence, for a comparison of the intrinsic strength of intermolecular binding in various dimers (see Section 3.2), the CCSD(T)/CBS Δ*E* values of the tilted T-shaped configuration were obtained as follows. The angles were kept fixed at $\vartheta_{A}=100{}^{\circ}$ and $\vartheta_{B}=13{}^{\circ}$, that is, at optimal values, after rounding them to 1° (see Table 1), and the distance R was varied over a very wide range, namely, from 440 to 900 pm. The resulting $\Delta E\left(R\right)$ curve thus covers separations from a repulsive region up to the dissociation into monomers. Figure 3 shows an accurate fit of this curve to a modified Dunham-type expansion, which is described by Equation (3) of Section 4. The curve reaches an Δ*E* of zero kJ/mol at the R distance of approximately 441.9 pm and has an inflexion point at around *R* = 542.1 pm where Δ*E* = −9.07 kJ/mol. The minimum of this curve is located at *R* = 492.7 pm, where Δ*E* = −11.80 kJ/mol. Importantly, these two values are very close to those found for an unconstrained minimum of the investigated dimer, which amounted to *R* = 493.1 pm where ΔE = −11.82 kJ/mol. This similarity of the predicted results shows that the constrained model should also be suitable for an analysis of differences in intermolecular binding between the tilted and canonical T-shaped configurations (see Section 3.1). Figure 3 thus compares the SAPT-DFT/CBS interaction energies to their CCSD(T)/CBS counterparts in the range from *R* = 460 to 700 pm. Clearly, the two data sets are very similar (all the values are listed in Table S2). Furthermore, an accurate fit of the SAPT-DFT/CBS results was obtained. It has a minimum and an inflexion point lying at around *R* values of 491.4 and 541.6 pm, respectively, in a close agreement with the aforementioned CCSD(T)/CBS results of 492.7 and 542.1 pm, respectively.

### 2.3. The Canonical T-Shaped Structure

**Figure 4:**
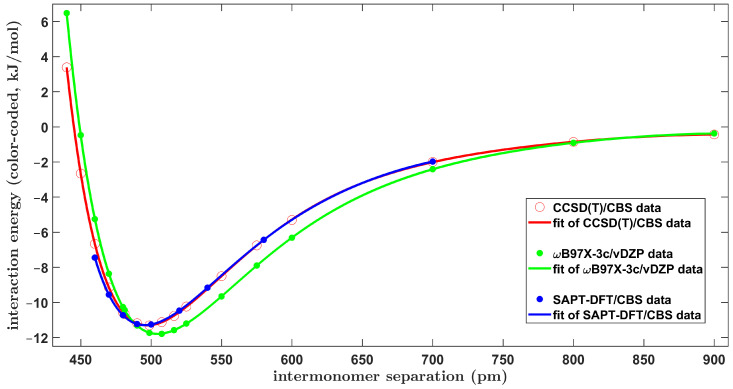
Plot of the distance dependence of the intermolecular interaction energy of the canonical T-shaped dimer of benzene.

In the rigid monomer approximation employed here, there is a single variable that defines the geometry of the *C*_2v_ symmetric T-shaped dimers of benzene, namely, the R distance. Figure 4 graphically presents the pertinent fit of the CCSD(T)/CBS Δ*E* as a function of this distance. As in the case of the tilted T-shaped configuration described in the preceding section, a very wide range of values of R was considered, and an analogous fit of the resulting curve was highly accurate, too. This $\Delta E\left(R\right)$ curve had a minimum for *R* of approximately 497.4 pm where Δ*E* = −11.30 kJ/mol, had a point of inflexion at *R* = 547.9 pm where Δ*E* = −8.66 kJ/mol and reached an ΔE of zero kJ/mol at *R* = 445.1 pm. Using these data as the benchmark, a performance of the computationally very cheap ωB97X-3c/vDZP method (see Section 2.2) was checked. All underlying values are collected in Table S3. For the CCSD(T)/CBS and ωB97X-3c/vDZP Δ*E* at 17 data points, the standard deviation of differences was 1.20 kJ/mol and the mean absolute difference was 0.87 kJ/mol. The maximal absolute difference was 3.09 kJ/mol and occurred for a point in the repulsive region, namely, where *R* = 440 pm (see Figure 4). The ωB97X-3c/vDZP $\Delta E\left(R\right)$ curve provided a qualitatively correct description of the investigated dimers. Specifically, the fitted curve featured a minimum around an *R* of 505 pm, with an Δ*E* of about −11.8 kJ/mol, and an inflexion point around an *R* of 559 pm, with an Δ*E* of about −9.0 kJ/mol. These values were only slightly higher than the aforementioned CCSD(T)/CBS results. Moreover, the curve reached an Δ*E* of zero kJ/mol at *R* = 449.2 pm, which is reasonably close to the reference *R* = 445.1 pm, and exhibited a monotonous decay of the predicted interaction energy with increasing *R* values (see Figure 4). The ωB97X-3c/vDZP method thus fared well in this challenging case.

Figure 4 also presents the SAPT-DFT/CBS data. They were, of course, computed at the same points from an interval of *R* ranging from 460 to 700 pm, as in the tilted T-shaped geometry and shown in Figure 3 above, as they are employed in Section 3.1 for a comparison of the two perpendicular-like configurations of the BD. Importantly, the SAPT-DFT/CBS values in this range of intermolecular distances closely match their CCSD(T)/CBS counterparts (see Figure 4 and Table S3). As a consequence, the minimum of the fitted SAPT-DFT/CBS $\Delta E\left(R\right)$ curve is located at *R* = 495.6 pm, where Δ*E* = −11.29 kJ/mol, which is in good accord with the corresponding CCSD(T)/CBS results.

### 2.4. The Parallel-Displaced Structure

**Figure 5:**
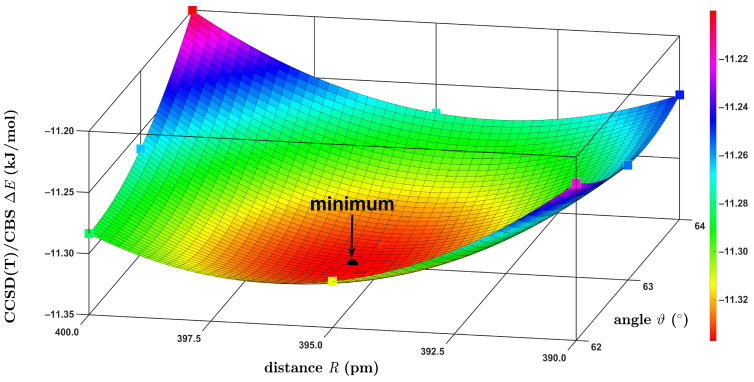
Surface plot of the intermolecular interaction energy in the region around a minimum of the slipped-parallel configuration of the benzene dimer.

In spite of the relatively small size of the BD, an accurate description of the SP configuration (see Figure 1) is known to be quite difficult for high-level quantum chemical methods. In particular, a ca. 0.4 kJ/mol difference in the Δ*E*, as predicted by the CCSD(T)/CBS and by precise QMC computations for the SP structure from the S66 set [47], was recently reported [28]. Here, the CCSD(T)/CBS minimum was found while employing two-dimensional (2D) grids that considered the aforementioned *R* distance and one of the $\vartheta_{A}$, $\vartheta_{B}$ angles. These angles had the same value due to the presence of the horizontal mirror plane in the SP configuration. Hence, the angular variable is denoted simply as ϑ in what follows. Scouting calculations were performed using the ωB97X-3c/vDZP method in order to inspect the sensitivity of the $\Delta E\left(R,\vartheta \right)$ results to changes in *R* and ϑ around the expected position of the minimum. Based on these results, an initial grid for the CCSD(T)/CBS computations was created. It covered points in quite wide $\langle 380;\;400\rangle$ pm and $\langle 151{}^{\circ};\;155{}^{\circ}\rangle$ intervals of the *R* and ϑ values, respectively. An inspection of the resulting $\Delta E\left(R,\vartheta \right)$ data enabled the choosing of a grid of only 3 × 3 points, which tightly enclosed a position of the minimum. Figure 5 presents the CCSD(T)/CBS Δ*E* obtained on this relatively narrow 2D grid (underlying values are provided in Table S4). These results illustrate the flat character of the PES around the minimum of the SP configuration, as the Δ*E* values computed on this grid cover an interval of about one-third of the kJ/mol. The minimum where Δ*E* = −11.34 kJ/mol was located by interpolation at the $\left(R,\vartheta \right)$ values, which are listed in Table 1. Also listed in Table 1 is a final Δ*E* value that was obtained for the minimum geometry by an application of the procedure expressed by Equation (2) (see Section 4).

**Figure 6:**
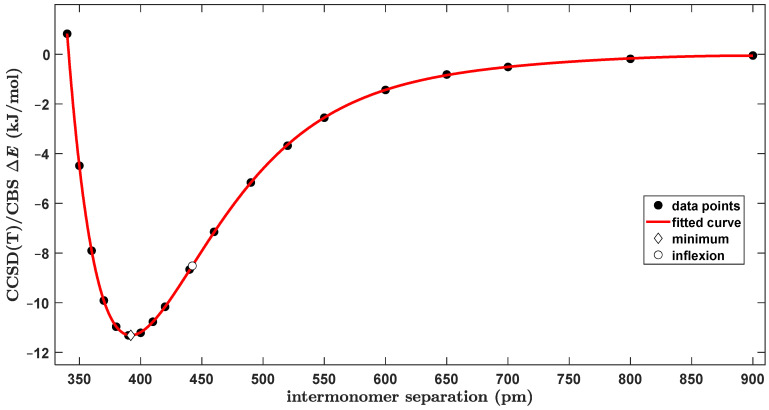
Plot of the distance dependence of the intermolecular interaction energy of the slipped-parallel dimer of benzene.

Furthermore, a series of constrained structures was prepared with ϑ fixed at 63.0° and covering a very large interval of values of the *R* distance, namely, from 340 to 900 pm. For these geometries, the CCSD(T)/CBS Δ*E* data were obtained in the same way as for the FT and TT configurations described above for the purpose of comparison of the intrinsic strength of intermolecular interactions in various binding modes of the BD. This comparison is provided in Section 3.2, while Figure 6 shows the fitted $\Delta E\left(R\right)$ curve (the underlying values are collected in Table S5). The curve reaches an Δ*E* of zero kJ/mol at 341.3 pm, has a minimum at *R* = 392.0 pm where Δ*E* = −11.33 kJ/mol and has an inflexion point at *R* = 442.2 pm where Δ*E* = −8.51 kJ/mol.

## 3. Discussion

### 3.1. The Origin of the Global Minimum

**Figure 7:**
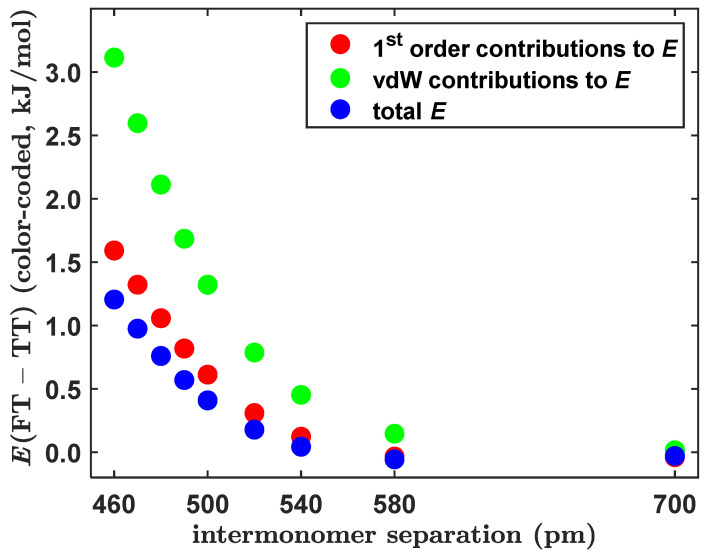
Plot of the distance dependence of the differences between predicted values of various components of the interaction energy of the tilted and canonical T-shaped configurations of the benzene dimer.

This revisit of the most stable configurations of the BD consistently applied the CCSD(T)/CBS methodology to obtain the minimum geometries and associated Δ*E* values of the TT, FT and PD structures. An ordering of the enthalpic stabilization of these structures agreed with the most recent results [10], as a matter of course, and the differences are insignificant between the respective Δ*E* values found here and in references [10,18] (see Table 1). Furthermore, the deformation energy of the monomers (see the most recent investigations [48,49], into an influence of internal deformations on the strength of noncovalent bonding) was found to be small and very similar in the TT and PD minima, amounting to 0.127 and 0.096 kJ/mol when estimated for the TT and PD minima, respectively, at the CCSD(T)/CBS level using the pertinent PBE0-D3/def2-TZVPP geometries (see Section 4 for details and references). The present structural and energetics data were utilized in the framework of an analysis of factors that caused the tilted T-shaped dimer to be the GM, and in the subsequent paragraph, they were employed for a description of the intrinsic strength of intermolecular interactions. The difference in the final Δ*E* values predicted for the CCSD(T)/CBS optimized geometries of the tilted and canonical T-shaped configurations amounted to −0.50 kJ/mol (see Table 1). An almost identical result of −0.51 kJ/mol was obtained from the DFT-SAPT/CBS interaction energy curves, which are presented in Section 2.2 and Section 2.3 and shown to closely match their CCSD(T)/CBS counterparts. Specifically, the DFT-SAPT/CBS interaction energy values in the minimum of the pertinent curve were −11.80 and −11.29 kJ/mol for the TT and FT configurations, respectively. The DFT-SAPT/CBS results are thus fully reliable and employed here to describe the physical contributions to the total value of the interaction energy in the two investigated structures (details of the underlying calculations, together with references, can be found in Section 4). It should be noted that in the rest of this section, the symbol *E* (and not Δ*E*) is used for various components of the DFT-SAPT/CBS interaction energy. Namely, $E_{elst}$ and $E_{exch}$ denote the electrostatic and exchange (Pauli repulsion) terms, respectively, arising in the first order of the perturbation theory of intermolecular interactions [50]. The sum of these two contributions is denoted as $E_{1}\;(E_{1}=E_{elst}+E_{exch}\;)$. Furthermore, $E_{disp}$ designates the sum of the second-order London dispersion terms, while $E_{ind}$ collects the second-order induction terms and an estimate of higher-order contributions. The total DFT-SAPT/CBS interaction energy, $E_{total}$, is the sum of the aforementioned components ($E_{total}=E_{1}+E_{disp}+E_{ind}$). Importantly, Carter-Fenk and Herbert proposed to group the $E_{exch}$ and $E_{disp}$ terms together to obtain what they termed the “van der Waals” (vdW) contribution to the interaction energy, $E_{vdW}=E_{exch}+E_{disp}$ [31]. Of course, $E_{vdW}=E_{total}-E_{elst}-E_{ind}$, and they used this partitioning in an analysis of intermolecular stacking interactions in the dimers of organic molecules [51] (this analysis was questioned in reference [52]). They also noticed that their $E_{vdW}$ data correctly predicted the TT configuration of the BD to be “slightly lower in energy than the canonical T-shaped structure” [31]. However, as the present DFT-SAPT/CBS results show (see Figure 7), a preference for the tilted structure can be much better explained by considering *E*_1_ contributions instead of their $E_{vdW}$ counterparts. This holds throughout a wide range of intermolecular separations, namely, from 460 to 700 pm (raw values of pertinent interaction energy terms are collected in Table S7). It should be pointed out that at lower distances between monomers, the model that employed the $E_{vdW}$ data significantly overestimated the differences between the total interaction energy of the FT and TT configurations, and this overestimation was much lower when the *E*_1_ values were considered. In a close vicinity of the respective minima, that is, at *R* = 490 pm, the difference between the $E_{total}$ values of the two perpendicular arrangements amounted to approximately −11.26 − (−11.67) = 0.41 kJ/mol, while the corresponding differences between the $E_{vdW}$ and *E*_1_ data were 1.32 and 0.62 kJ/mol, respectively. These results imply that for a given *E* distance, the $\left(E_{disp}+E_{ind}\right)$ sum has a similar value in the two investigated configurations, contrary to the corresponding $\left(E_{exch}+E_{disp}\right)$ sum (see Table S7). Due to this partial cancellation of the higher-order contributions to the interaction energy, changes in the first-order terms quantitatively explain the observed preference for the tilted structure to be the GM.

### 3.2. The Intrinsic Strength of Intermolecular Interactions

**Figure 8:**
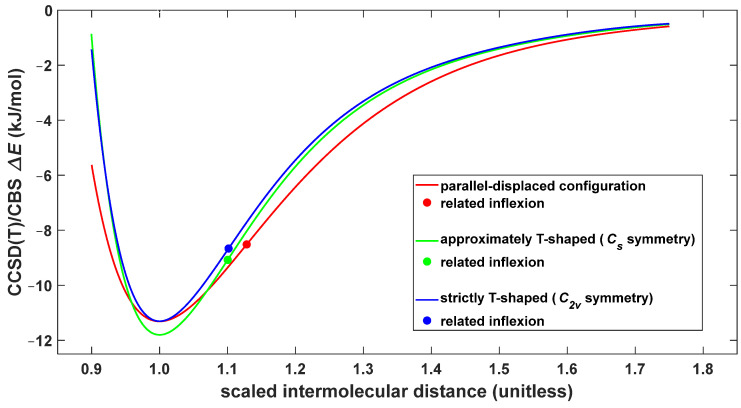
Plot of the intrinsic strength of intermolecular interactions in three configurations of the benzene dimer.

The CCSD(T)/CBS results presented above can be usefully employed for an analysis of the intrinsic strength of intermolecular interactions in the three investigated dimers. Using accurate fits of the respective CCSD(T)/CBS $\Delta E\left(R\right)$ curves, which are shown in red in Figure 3, Figure 4 and Figure 6, the dependence of the Δ*E* upon the relative distance, *r*, can be immediately obtained. This distance is defined as $r=R/R_{min}$, where $R_{min}$ is a position of the minimum found for the pertinent $\Delta E\left(R\right)$ curve. Obviously, the resulting $\Delta E\left(r\right)$ curves remove a direct effect upon the Δ*E* values of different separations between the centers of mass of the monomers in the cofacial and perpendicular arrangements of the BD. This way, the Δ*E* data of various spatial orientations are comparable in all investigated intervals of the original distance, *R*, and the intrinsic strength of concomitant intermolecular interactions can be compared between the TT, FT and PD structures. Figure 8 shows the $\Delta E\left(r\right)$ curves for relative distances between 0.90 and 1.75. Importantly, only in a narrow region around the minima, namely, for values of *r* ranging from approximately 0.96 to 1.07, the T-shaped configurations are intrinsically more stable than the parallel-displaced geometry. This indicates a preference for the π∙∙∙π stacking interaction between phenyls in irregular structures: for instance, disordered regions of proteins [53]. However, at a given nonzero temperature, the total strength of nonbonding interactions between dimers is affected by factors like vibrational energy and nuclear quantum effects, which may be substantially different among dimeric geometries [33]. This is clearly important for the investigated configurations of the BD, as the differences in their Δ*E* data are small in terms of absolute values (several kJ/mol or less). Nevertheless, Figure 8 shows that in compressed geometries (that is, for *r* values of lower than about 0.95), the π∙∙∙π stacking has a much higher intrinsic strength than the C–H∙∙∙π interaction, and that the Δ*E* values of all configurations dropped below 1 kJ/mol at values of the reduced distance higher than ca. 1.60.

## 4. Materials and Methods

The CP CCSD(T)/CBS interaction energy for the PES searches was evaluated using Equation (1):

(1) $${\Delta E}_{CCSD\left(T\right)}^{CBS}={\Delta E}_{HF}^{a5Z}+{\Delta E}_{MP2}^{a5Z}+{\Delta E}_{post-MP2}^{aTZ},$$

where the subscripts denote the respective energy terms, namely, the total Hartree–Fock energy (“HF”), the MP2 correlation energy (“MP2”) and the higher-order correlation energy (“post-MP2”), and the superscripts specify the augmented correlation-consistent polarized-valence basis set [37,38] that was used to compute the respective term. The final CP CCSD(T)/CBS interaction energy of the optimized structures was obtained as the sum of the estimates of the HF energy and the total correlation energy:

(2) $${\Delta E}_{CCSD\left(T\right)}^{CBS}={\Delta E}_{HF}^{a5Z}+{\Delta E}_{corr.}^{aTZ\to aQZ},$$

where the subscript “HF” denotes the total HF energy contribution as in Equation (1), the subscript “corr.” denotes the total correlation energy contribution and the right arrow indicates an application of the two-point extrapolation formula from reference [54] to pertinent correlation energies. The HF/a5Z and MP2/a5Z energies were computed in Turbomole version 7.1 [55]. The MP2/a5Z correlation energies were obtained in the resolution-of-the-identity integral approximation [56,57] while the relevant auxiliary basis sets were applied [57]. Calculations of all the CCSD(T) energies were carried out in Molpro 2022.2 [58].

The density-fitting variant of the SAPT-DFT method [59] was used as implemented in Molpro 2022.2 The computational procedures described in our recent work [60], were adopted in order to estimate the respective SAPT-DFT/CBS terms. In brief, the $E_{elst}$, $E_{exch}$, $E_{disp}$ and $E_{ind}$ contributions to the total interaction energy, $E_{total}$, from Section 3.1 are related to the underlying interaction energy terms as follows: $E_{elst}$ and $E_{exch}$ are the electrostatic polarization and Pauli exchange energy contributions, respectively, arising in the first order of the perturbation theory of intermolecular interactions [61]; $E_{disp}$ is the London dispersion energy contribution obtained as the sum of the second-order terms $E_{disp.}^{SAPT\;(2)}$ and $E_{disp.-exch.}^{SAPT\;(2)}$ [62]; and $E_{ind}$ is the induction energy contribution approximated by the sum of the second-order terms $E_{ind.}^{SAPT\;(2)}$ and $E_{ind.}^{SAPT\;(2)}$ [63] and the correction term $E_{\delta (HF)}^{SAPT\;}$, which is calculated at the HF level [64].

The deformation energy was computed as the difference between the total CCSD(T)/CBS energy of the respective monomers of an investigated dimer and two isolated benzene molecules. The related CCSD(T)/CBS energies were estimated using Equation (1). The PBE0-D3/def2-TZVPP approach (the PBE0 hybrid functional [65] applied together with the D3 empirical dispersion correction [66] and the triple zeta valence basis set from reference [67]) was used to locate the PES minimum. The default algorithms and settings of the Gaussian 16 suite of codes [68] were used.

An estimation of the ωB97X-3c/vDZP interaction energy was carried out in the ORCA 5.0.3 program package [69]. The ORCA input files were created using the “o4wb3c.f” code downloaded from GitHub [70].

The $\Delta E\left(R\right)$ curves were obtained by the least-squares fit of the interaction energy data to the functional form expressed by Equation (3):

(3) $$\Delta E\left(R;\;r_{e},a_{0},a_{1},a_{2},a_{3},a_{4},a_{5},a_{6},V_{e}\right)=a_{0}\xi^{2}\left(1+a_{1}\xi +a_{2}\xi^{2}+a_{3}\xi^{3}+a_{4}\xi^{4}+a_{5}\xi^{5}+a_{6}\xi^{6}\right)+V_{e},$$

where $\xi =\left(R-r_{e}\right)/R$ and *R* is the distance between the centers of mass of the monomers. The trust region-reflective algorithm from the “lsqcurvefit” function of MATLAB^®^ Optimization Toolbox™ was applied, and a solution was then checked using the “e04ggf” subroutine from the NAG^®^ numerical library.

The 3D gridded Δ*E* data were interpolated by applying the cubic method of the “interp3” function of MATLAB^®^.

## 5. Conclusions

In this work, the interaction energies of three of the most enthalpically stable configurations of the BD were revisited by means of CCSD(T)/CBS computations performed for the CCSD(T)/CBS geometries, which were located with extensive searches of the PES. The results were in line with the most recent data [10] and served as benchmarks for the SAPT-DFT/CBS computational protocol. This protocol was found to be fully reliable. Hence, it was used to interpret the origin of structural preference for the GM, namely, the differences in individual contributions to the interaction energy of the tilted and canonical T-shaped structures. This led to the first major finding of the present study: the sum of the electrostatics and Pauli repulsion terms arising in the first order of the perturbation theory of intermolecular interactions quantitatively explains the well-known preference for tilting in perpendicular-like arrangements of the BD. Furthermore, the CCSD(T)/CBS data were used to compare the intrinsic strength of intermolecular interactions in various parts of the dissociation curves of the investigated geometries. This comparison led to the second major finding of the present study: the π∙∙∙π stacking in regions further apart from a minimum should be expected to be more enthalpically favorable than the C–H∙∙∙π binding mode. Moreover, the CCSD(T)/CBS results of this investigation provide a firm ground for studying various properties of the BD [71] and for the testing of more approximate methods: for instance, the promising QMC variant [72]. These results could also be useful in the quickly evolving field of machine learning [73] and other data-driven models in computational chemistry.

## Figures and Tables

**Table 1:** The key geometrical parameters and pertinent intermolecular interaction energies of the three investigated configurations of the benzene dimer.

Geometry (Symmetry Group)	Parameter			
	R/pm	$\vartheta_{A}$/deg.	$\vartheta_{B}$/deg.	$\left|\Delta E\right|$/kcal/mol
Tilted T-shaped (*C*_s_)	493.1 ^a^	100.1 ^a^	12.6 ^a^	11.84 ^a^
	494.4 ^b^	99.22 ^b^	11.75 ^b^	11.67 ^b^
	492.4 ^c^	99.19 ^c^	11.09 ^c^	(11.83 ± 0.04) ^d^
Canonical T-shaped (*C*_2v_)	497.4 ^a^	—	—	11.34 ^a^
	497.0 ^b^ 495.0 ^e^			11.35 ^b^ (11.46 ± 0.13) ^d^
Slipped-parallel (*C*_2h_)	395.4 ^a^	62.8 ^a^	62.8 ^a^	11.21 ^a^
	393.7 ^b^ 384.0 ^e^	62.12 ^b^ 63.85 ^e^	62.12 ^b^ 63.85 ^e^	11.24 ^b^ (11.09 ± 0.08) ^d^

^a^ The CCSD(T)/CBS value obtained in this work; ^b^ The “pot3” value (see the text) from reference [18]; ^c^ The SCS-MP2/VTZ value from reference [10]; ^d^ The CCSD(T)/CBS value from reference [10]; ^e^ The CCSD(T)/VTZ value from reference [10].

## Data Availability

The data presented in this study are available in the article and in the Supplementary Materials.

## Supplementary Materials

The following supporting information can be downloaded at: https://www.mdpi.com/article/10.3390/ijms25158272/s1.
