# Supplementary material for: Revisiting the Most Stable Structures of the Benzene Dimer
Source: Int J Mol Sci. 2024 Jul 29;25(15):8272. doi: 10.3390/ijms25158272 (PMC11312267; doi:10.3390/ijms25158272)
Supplement: Supplementary file 1 [file ijms-25-08272-s001.zip › ijms-3105440-supplementary.pdf]

Supplementary Information to "Revisiting the most stable structures of the benzene dimer" by Czernek & Brus (*Int. J. Mol. Sci.* 2024)

Table of Contents

Figure S1 at page S2: plot of the ωB97X-3c/vDZP $\Delta E(\beta_A, \gamma_B, R)$ data;

Figure S2 at page S3: plot of the CCSD(T)/CBS $\Delta E(\beta_A, \gamma_B, R)$ data;

Table S1 at page S4: listing of the CCSD(T)/CBS $\Delta E(\beta_A, \gamma_B, R)$ data;

Table S2 at page S5: data for Figure 3;

Table S3 at page S6: data for Figure 4;

Table S4 at page S7: data for Figure 5;

Table S5 at page S8: data for Figure 6;

Table S6 at page S9: data for Figure 7.

Figure S1. Plot of the ωB97X-3c/vDZP $\Delta E(\beta_A, \gamma_B, R)$ data of the TT structure of the BD. Also visualized are slice planes of the interpolated values for R = 500.00 pm and γ_B = 256.12°, and a point of the $\Delta E(\beta_A, \gamma_B, R)$ minimum (at this point, these two planes intersect a slice plane obtained for β_A = 169.71°, which is not shown).

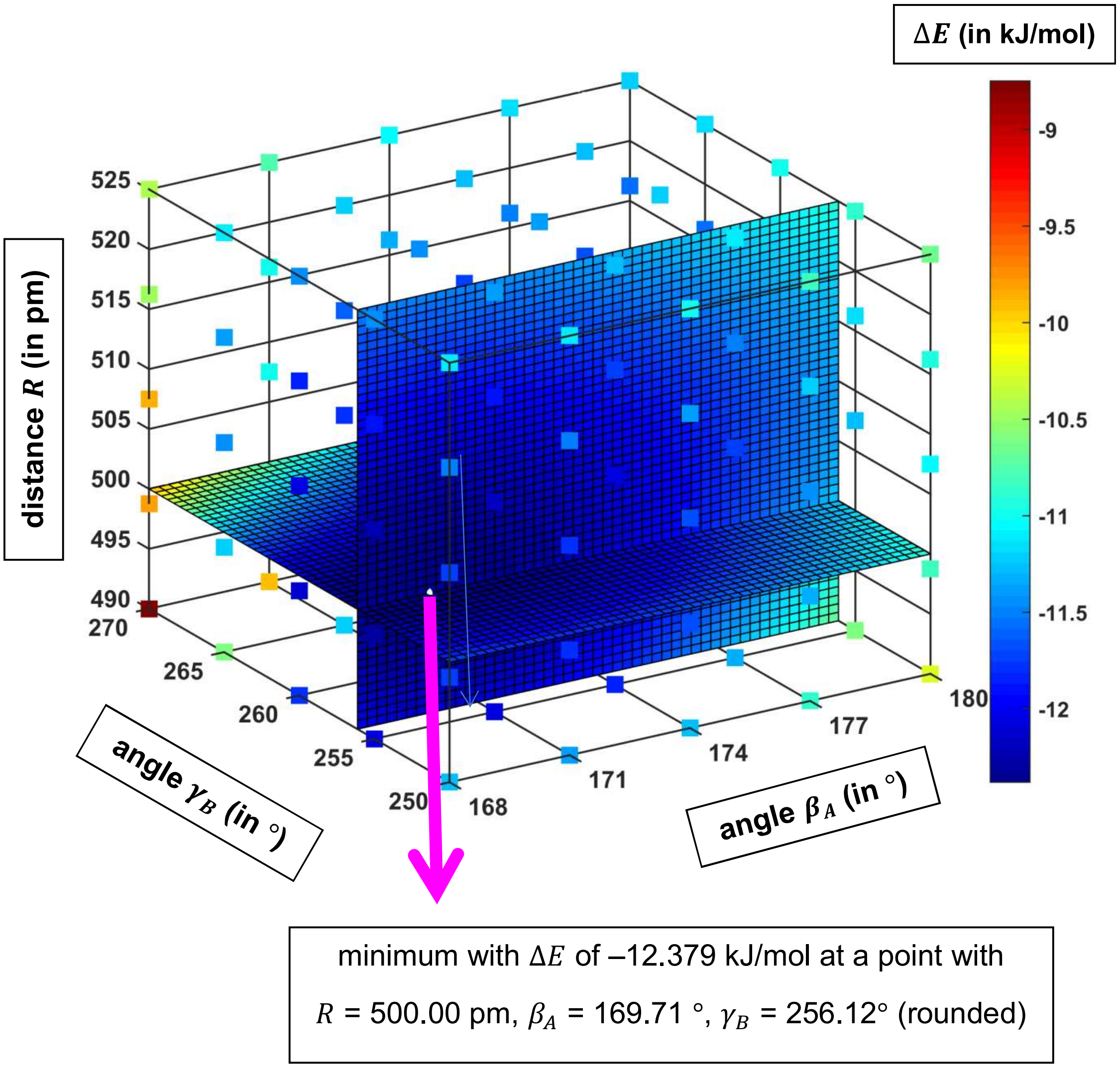

Figure S2. Plot of the CCSD(T)/CBS $\Delta E(\beta_A, \gamma_B, R)$ data of the TT structure of the BD. Also shown is a cut through the interpolated data for a value of R in the $\Delta E(\beta_A, \gamma_B, R)$ minimum.

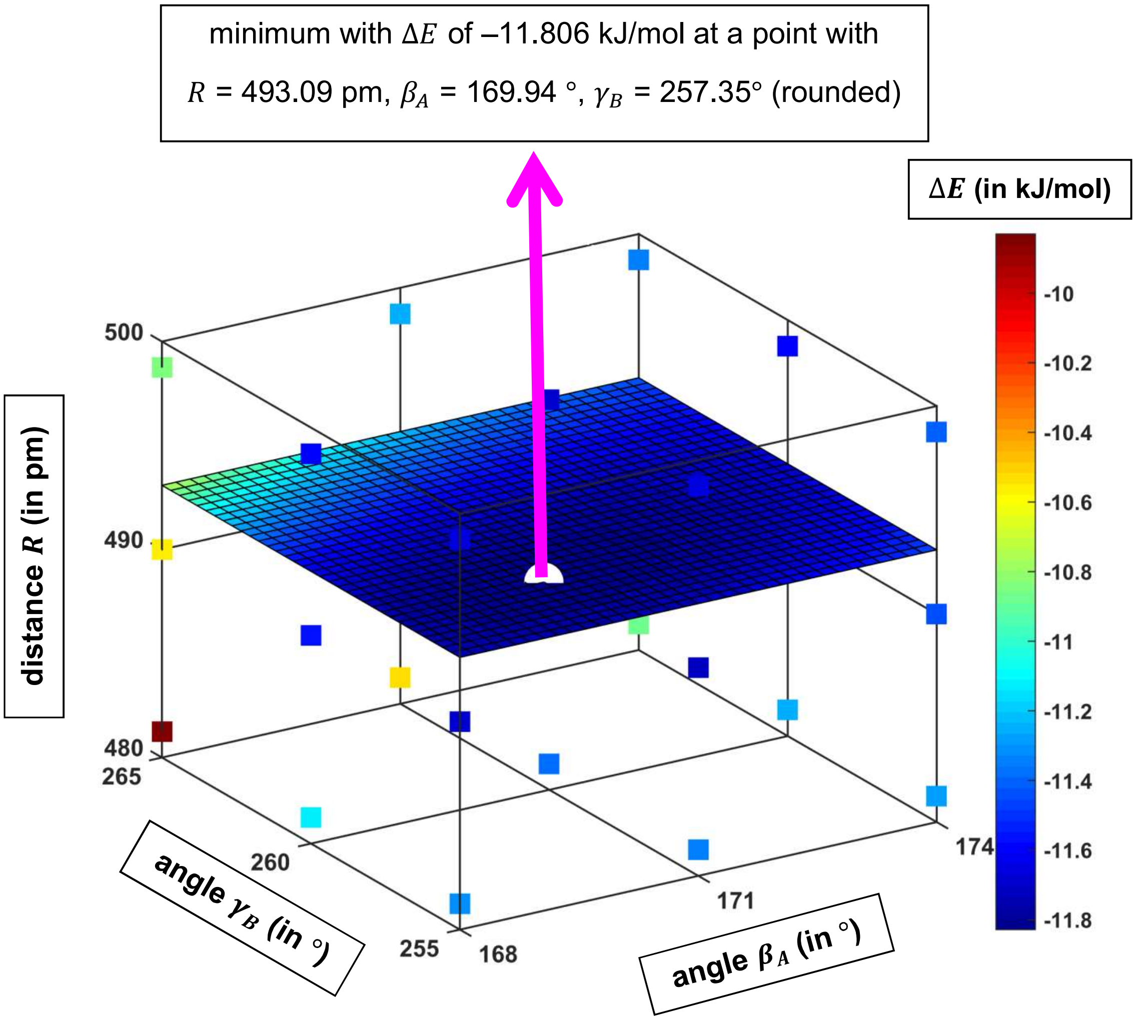

Table S1. The CCSD(T)/CBS $\Delta E(\beta_A, \gamma_B, R)$ data of the TT structure of the BD as actually used to create Figure S2.

β_A (in °)	γ_B (in °)	R (in pm)	ΔE (in kJ/mol)
168.0	255.0	481.25	–11.3187
168.0	255.0	490.00	–11.7012
168.0	255.0	498.75	–11.6449
168.0	260.0	481.25	–11.1366
168.0	260.0	490.00	–11.5549
168.0	260.0	498.75	–11.5984
168.0	265.0	481.25	–9.8303
168.0	265.0	490.00	–10.5680
168.0	265.0	498.75	–10.8345
171.0	255.0	481.25	–11.3406
171.0	255.0	490.00	–11.7038
171.0	255.0	498.75	–11.6537
171.0	260.0	481.25	–11.3824
171.0	260.0	490.00	–11.7235
171.0	260.0	498.75	–11.6916
171.0	265.0	481.25	–10.5329
171.0	265.0	490.00	–11.1063
171.0	265.0	498.75	–11.2448
174.0	255.0	481.25	–11.2687
174.0	255.0	490.00	–11.4432
174.0	255.0	498.75	–11.3997
174.0	260.0	481.25	–11.2651
174.0	260.0	490.00	–11.6240
174.0	260.0	498.75	–11.6076
174.0	265.0	481.25	–10.8724
174.0	265.0	490.00	–11.3602
174.0	265.0	498.75	–11.3381

Table S2. Data actually used for fitting of the $\Delta E(R)$ curves of the TT configuration of the BD. Resulting curves are shown in Figure 3. All values of the distance and interaction energy are given in pm and kJ/mol, respectively.

CCSD(T)/CBS data points		SAPT-DFT/CBS data points	
R	ΔE	R	ΔE
440.00	1.304863638573650	460.00	–8.653276065283649
450.00	–4.423416092650001	470.00	–10.531071661235812
460.00	–8.065218687242648	480.00	–11.488101512042029
470.00	–10.240399590829412	490.00	–11.797785764602702
480.00	–11.455140828403074	500.00	–11.666255209786218
490.00	–11.795164933048881	520.00	–10.646624857142976
500.00	–11.716479181258620	540.00	–9.203980521732028
510.00	–11.286737724550406	580.00	–6.379696824334053
520.00	–10.673928945663491	700.00	–1.977202438030135
530.00	–9.929898716324473		
540.00	–9.259013483781260		
560.00	–7.750172187609806		
580.00	–6.366374023006244		
620.00	–4.262838690471455		
660.00	–2.848100538928279		
700.00	–1.954407176892772		
750.00	–1.266301121543734		
800.00	–0.862218912778445		
900.00	–0.389568250519375		

Table S3. Data actually used for fitting of the $\Delta E(R)$ curves of the FT configuration of the BD. Resulting curves are shown in Figure 4. All values of the distance and interaction energy are given in pm and kJ/mol, respectively.

CCSD(T)/CBS data points		ωB97X-3c/vDZP data		SAPT-DFT/CBS data points	
R	ΔE	R	ΔE	R	ΔE
440.00	3.393278406919990	440.00	6.480864616461467	460.00	–7.448152468673920
450.00	–2.653643327868949	450.00	–0.469740248158971	470.00	–9.556387318309595
460.00	–6.660969629115911	460.00	–5.253791969837565	480.00	–10.728624925154730
470.00	–9.144818716029391	470.00	–8.366155642354208	490.00	–11.228566222548380
480.00	–10.579609932356050	480.00	–10.263103476071208	500.00	–11.257101806355811
481.25	–10.662378291978200	481.25	–10.434354667227915	520.00	–10.468529283001622
490.00	–11.182090779168234	490.00	–11.310411915760575	540.00	–9.160976608556894
498.75	–11.304851960172186	498.75	–11.733257901000869	580.00	–6.436601272698920
507.50	–11.123176598988934	507.50	–11.787578500963164	700.00	–2.007412039927972
516.25	–10.771262744546888	516.25	–11.579233224456495		
525.00	–10.232669034391591	525.00	–11.199337019185386		
550.00	–8.502779933238216	550.00	–9.652076765826756		
575.00	–6.751869466964237	575.00	–7.901147243574727		
600.00	–5.303079444086222	600.00	–6.312790046574193		
700.00	–1.997705350289152	700.00	–2.412635959696026		
800.00	–0.856929706589028	800.00	–0.907874393888441		
900.00	–0.430933940391498	900.00	–0.375091332855710		

Table S4. The CCSD(T)/CBS $\Delta E(R, \vartheta)$ data of the PD structure of the BD as actually used to create Figure 5.

R (in pm)	ϑ (in °)	ΔE (in kJ/mol)
390.0	152.0	–11.2212
395.0	152.0	–11.2562
400.0	152.0	–11.2482
390.0	153.0	–11.3122
395.0	153.0	–11.3347
400.0	153.0	–11.2736
390.0	154.0	–11.2836
395.0	154.0	–11.2639
400.0	154.0	–11.2002

Table S5. Data actually used for fitting of the CCSD(T)/CBS $\Delta E(R)$ curve of the PD configuration of the BD. The resulting curve is shown in Figure 6. All values of the distance and interaction energy are given in pm and kJ/mol, respectively.

R	ΔE
340.0	0.825079689886579
350.0	–4.485804365460680
360.0	–7.905833427803131
370.0	–9.911666939928551
380.0	–10.963189832324099
390.0	–11.312177577813316
400.0	–11.211704135150512
410.0	–10.765419948985283
420.0	–10.161938497722346
440.0	–8.660805960987503
460.0	–7.147907298649116
490.0	–5.161388355754937
520.0	–3.679306546947089
550.0	–2.553776627630853
600.0	–1.437052154979014
650.0	–0.814065764615279
700.0	–0.510139911700364
800.0	–0.182989699268276
900.0	–0.049446033549188

Table S6. The SAPT-DFT/CBS interaction energy terms at various intermonomer separations of the FT and TT (given in parentheses) configurations of the BD. Differences between the corresponding FT and TT values are plotted in Figure 7.

R (in pm)	E_1 (in J/mol)	E_{vdW} (in J/mol)	E_{total} (in J/mol)
460.00	29509<br>(27918)	16620<br>(13505)	–7448<br>(–8653)
470.00	21791<br>(20469)	9225<br>(6628)	–9556<br>(–10531)
480.00	15920<br>(14863)	3988<br>(1875)	–10729<br>(–11488)
490.00	11481<br>(10663)	368<br>(–1316)	–11229<br>(–11798)
500.00	8145<br>(7533)	–2053<br>(–3375)	–11257<br>(–11666)
520.00	3803<br>(3494)	–4513<br>(–5300)	–10469<br>(–10647)
540.00	1446<br>(1324)	–5138<br>(–5591)	–9161<br>(–9204)
580.00	–393<br>(–358)	–4321<br>(–4467)	–6437<br>(–6380)
700.00	–577<br>(–537)	–1345<br>(–1361)	–2007<br>(–1977)
